# Supplementary material for: Castor is a temporal transcription factor that specifies early born central complex neuron identity
Source: Development. 2024 Dec 16;151(24):dev204318. doi: 10.1242/dev.204318 (PMC11701512; doi:10.1242/dev.204318)
Supplement: Supplementary information [file develop-151-204318-s1.pdf]

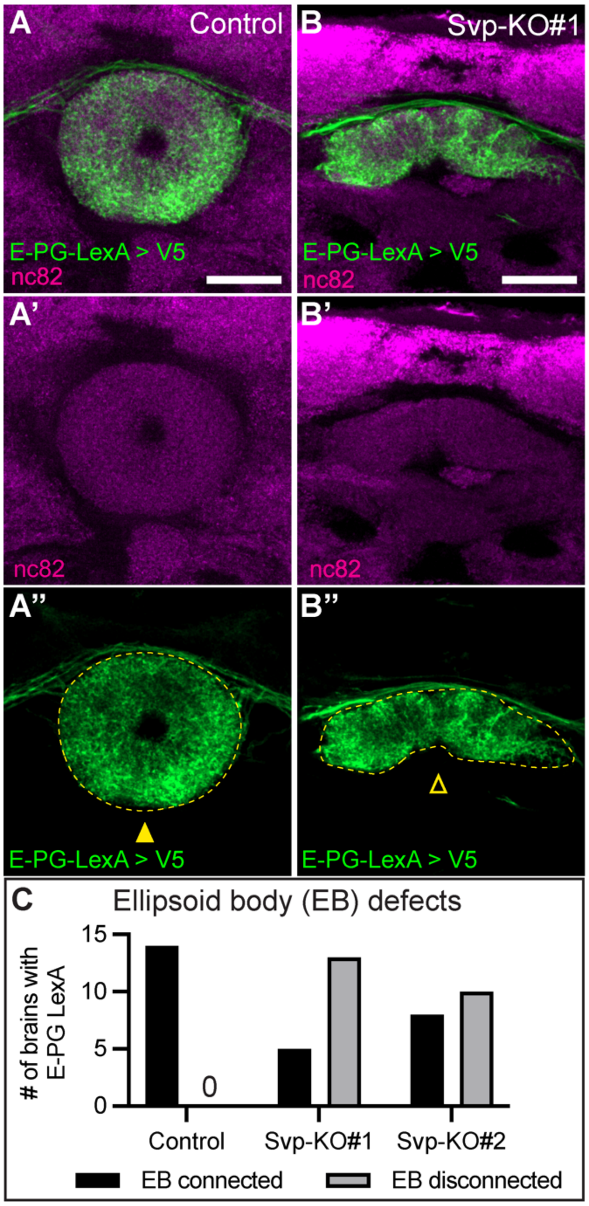

Fig. S1. Loss of Seven-up in Type 2 neuroblasts leads to altered E-PG ellipsoid body morphology.

(A-B) Control (A) and Seven-up (Svp) knockout (B). There is altered morphology of R60D05-LexA+ E-PG neurons in the Svp knockout with a disconnected ellipsoid body (EB). (C) Quantification. Control, $n = 14$; Svp-KO#1, $n = 18$; Svp-KO#2, $n = 18$. P-value determined by Chi-square analysis ** $P<0.001$. In all images, LexA+ neurons driving V5 expression are in green, neuropil nc82 in magenta, and EB outlined in yellow dashed line. Closed arrowhead indicates a connected EB; open arrowhead indicates a disconnected EB. Scale bars: 20 µm.

Development • Supplementary information

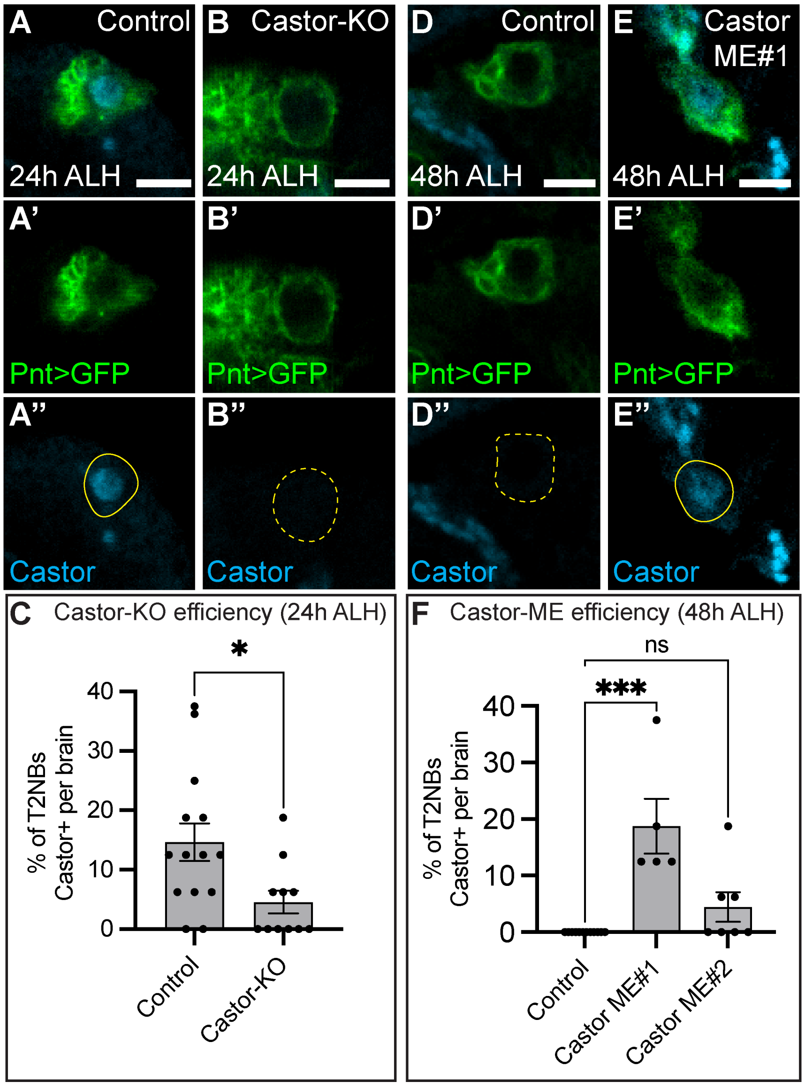

Fig S2. Generating Type 2 lineage specific Castor knockout and misexpression lines.

(A-C) Control (A) and Castor-KO (B) show a loss of Castor in Type 2 neuroblasts (T2NB) in the Castor-KO at 24h after larval hatching (ALH). (C) Quantification. Bar plot shows mean with standard error of the mean (SEM). Each dot represents one brain. Control, *n*=14; Castor-KO, *n*=11. *P*-value was determined using an unpaired *t*-test, **P*=0.017. (D-F) Control (A) and Castor misexpression (ME) (B) show ectopic expression of Castor in Type 2 neuroblasts (T2NB) in the Castor-ME at 48h after larval hatching (ALH). (F) Quantification. Bar plot shows mean with SEM. Each dot represents one brain. Control, *n*=12; Castor 2nd, *n*=5; Castor 3rd, *n*=7. *P*-values were determined using a one-way ANOVA, ***P*<0.001, followed by Dunnett's test between the control and Castor-MEs: Control versus Castor ME#1, ****P*<0.001; Control versus Castor ME#2, ns *P*=0.24. In all images, Pnt-Gal4 driving GFP in green and T2NBs outlined in yellow; solid line indicates positive for Castor, dashed line indicates negative for Castor; Castor, cyan. Scale bars: 5 μm.

Development • Supplementary information

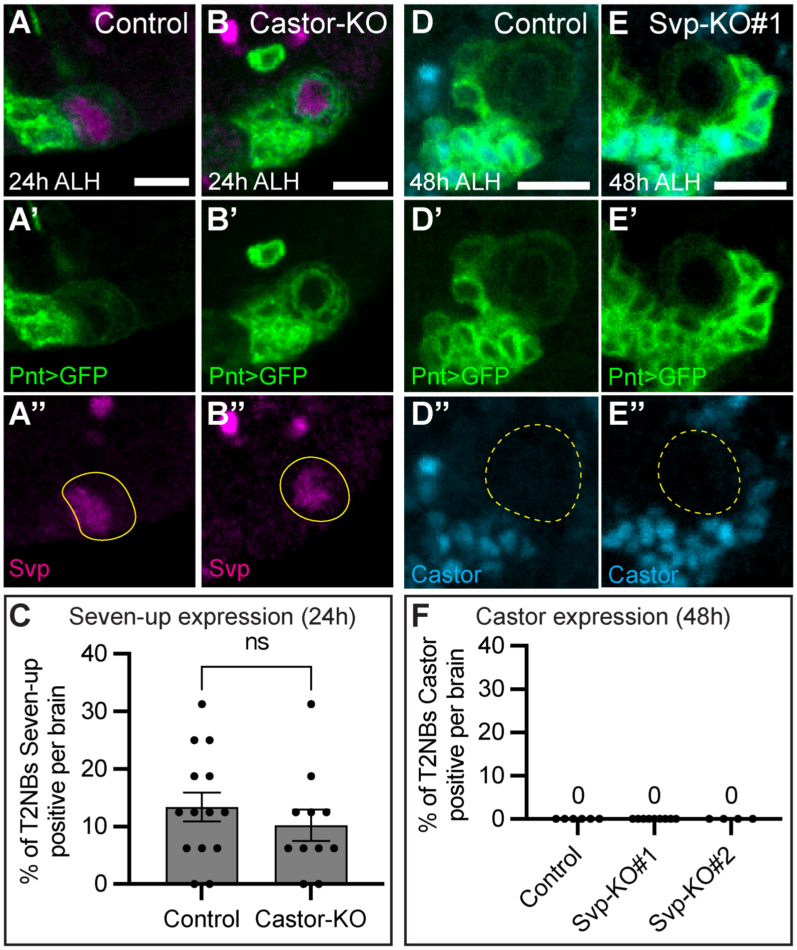

Fig. S3. Castor and Seven-up do not cross regulate in Type 2 neuroblasts.
(A-C) Control (A) and Castor-KO (B) shows no change in Seven-up (Svp) expression in Type 2 neuroblasts (T2NB) in the Castor-KO at 24 h ALH. (C) Quantification. Bar plot shows mean with standard error of the mean. Each dot represents one brain. Control, *n*=14; Castor-KO, *n*=11. *P*-value was determined using an unpaired *t*-test, **P*=0.40. (D-E) Control (D) and Svp-KO#1 (E) shows no extended expression of Castor in Type 2 neuroblasts (T2NB) in the Svp-KO at 48 h ALH. (F) Quantification. Bar plot shows mean with no error bars due to no differential values. Each dot represents one brain. Control, *n*=6; Svp-KO#1, *n*=9; Svp-KO#2, *n*=4. *P*-value was not determined due to no differential values reported between conditions. In all images, Pnt-Gal4 driving GFP in green and T2NBs outlined in yellow; solid line indicates positive for transcription factor of interest, dashed line indicates negative for transcription factor of interest; Svp, magenta; Castor, Cyan. Scale bars: 5 µm.

Development • Supplementary information

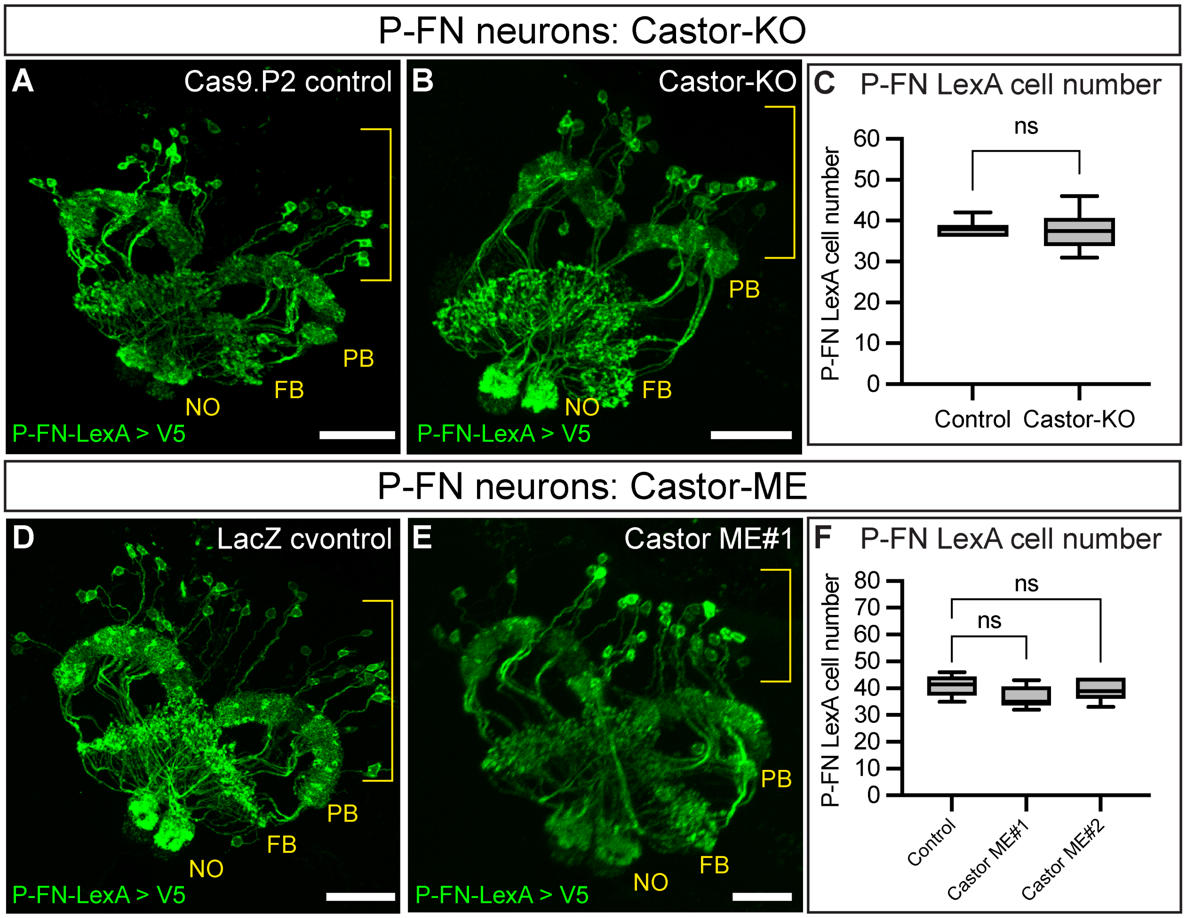

Fig. S4. Castor is not necessary or sufficient to specify late born P-FN adult neuron identities.

(A-C) Cas9.P2 control (A) and Castor-KO (B) shows no change in R16D01-LexA+ P-FN neurons. Brackets indicate cell body region; yellow text labels central complex neuropils: protocerebral bridge (PB), fan-shaped body (FB), and noduli (NO). (C) Quantification. Box and whisker plots display the minimum and maximum range of the data with interquartile range. Control, n=7; Castor-KO, n=8. P-value was determined using an unpaired t-test, ns P=0.96. (D-F) LacZ control (D) and Castor ME#1 (E) shows no change in R16D01-LexA+ P-FN neurons. (F) Quantification. Box and whisker plots display the minimum and maximum range of the data with interquartile range. Control, n=6; Castor ME#1, n=6; Castor ME#2, n=7. P-value was determined using a one-way ANOVA, P=0.20, followed by Dunnett's test between the control and Castor misexpressions: Control versus Castor ME#1, ns P=0.14; Control versus Castor ME#2, ns P=0.73. In all images, LexA+ neurons driving membrane-bound V5 are in green. Scale bars: 20 μm.

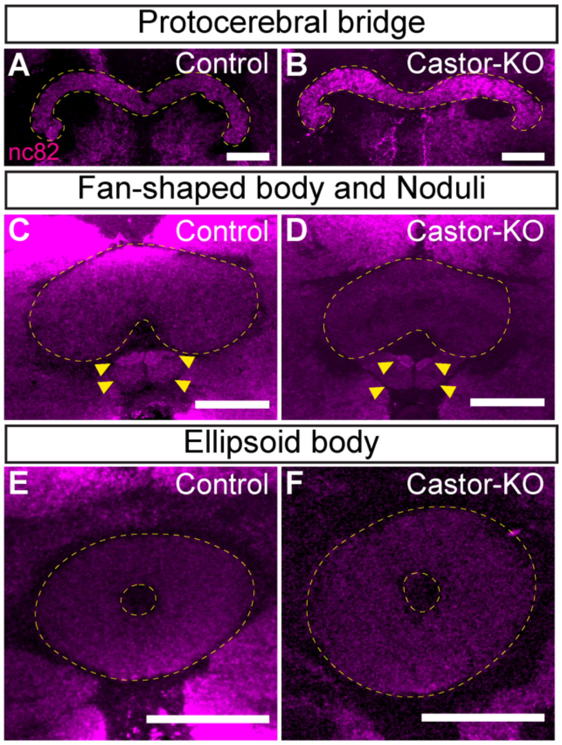

Fig. S5. Castor is not necessary for the formation of the Central Complex neuropils. (A-F) Neuropil stains of the CX neuropils in Cas9.P2 control (A, C, E) and Castor-KO (B, D, F) shows no major change in the PB (A-B), FB and NO (C-D), or EB (E-F) neuropils. In all images, nc82 expression is in magenta and dashed yellow lines indicate the neuropil boundaries. Yellow arrowheads indicate NO neuropils (C-D). Scale bars: 20 µm.

Table S1. Transgenes and *Drosophila melanogaster* stock lines used.

Genotype	Source and identifier	Additional information
R12D09-LexA	BDSC #54419	Expressed in P-EN neurons
R60D05-LexA	BDSC #52867	Expressed in E-PG neurons
R16D01-LexA	BDSC #52503	Expressed in P-FN neurons
13xLexAop-myr::GFP	BDSC #32210	Expresses membrane bound GFP under LexAop control
10XUAS-IVS-myr::GFP	BDSC #32198	Expresses membrane bound GFP under UAS control
Pointed-Gal4	*PMID:* 22143802 *14-94*	Expressed in Type 2 lineage starting in the neuroblast
10xUAS-IVS-myr::smGdP::HA, 13xLexAop2-IVS-myr::smGdP::V5	BDSC #64092	Expresses HA membrane tag under UAS control, V5 membrane tag under LexAop control
hsFLP; ;UAS-Cas9.P2	BDSC #58986	Expresses Cas9 under UAS control
hsFLP; UAS-sgRNA::svp ;	VDRC #341527	Expresses two short guide RNAs against Svp under UAS control; Svp-KO#1
hsFLP; UAS-sgRNA::svp ;	VDRC #341390	Expresses two short guide RNAs against Svp under UAS control; Svp-KO#2
hsFLP; UAS-sgRNA::castor ;	VDRC #341386	Expresses two short guide RNAs against Castor under UAS control
; UAS-LacZ ;	BDSC #8529	Expresses LacZ under UAS control
; UAS-Castor ;	Odenwald, W.	Expresses Castor under UAS control
; ; UAS-Castor	Odenwald, W.	Expresses Castor under UAS control

Table S2. Genetic crosses for each experiment.

Figures	Summary	Genetic cross
Figure 1C-E	Labels adult E-PG neurons for EdU birth dating	Females containing *13xLexAop-myr::GFP* were crossed to males containing R60D05-*LexA*
Figure 2A-C<br>Figure 4M-O	Labels adult E-PG (i) and P-EN (ii) neurons for molecular markers	Self-cross of females and males containing (i) *10xUAS-IVS-myr::smGdP::HA, 13xLexAop2-IVS-myr::smGdP::V5*; *R60D05-LexA*; *Pointed-Gal4* or (ii) *10xUAS-IVS-myr::smGdP::HA, 13xLexAop2-IVS-myr::smGdP::V5* ; *R12D09-LexA* ; *Pointed-Gal4*
Figure 3A-F<br>Figure S1A-C	Labels adult E-PG neurons and drives UAS constructs in the Type 2 progenitors for:<br>Cas9.P2 control (i)<br>Svp-KO#1 (ii)<br>Svp-KO#2 (iii)	Females containing *10xUAS-IVS-myr::smGdP::HA, 13xLexAop2-IVS-myr::smGdP::V5*; *R60D05-LexA*; *Pointed-Gal4* were crossed to males containing either (i) *; ; UAS-Cas9.P2*, (ii) ; *UAS-sgRNA::svp* (VDRC #341527) ; *UAS-Cas9.P2* , or (iii) ; *UAS-sgRNA::svp* (VDRC #341390) ; *UAS-Cas9.P2*
Figure 4A-L	Labels larval Type 2 lineage	Self-cross of females and males containing *10XUAS-IVS-myr::GFP ; Pointed-Gal4*
Figure 5A-F<br>Figure S5	Labels adult P-EN neurons and drives UAS constructs in the Type 2 progenitors for:<br>Cas9.P2 control (i)<br>Castor-KO (ii)	Females containing *10xUAS-IVS-myr::smGdP::HA, 13xLexAop2-IVS-myr::smGdP::V5*; *R12D09-LexA*; *Pointed-Gal4* were crossed to males containing either (i) *; ; UAS-Cas9.P2*, (ii) ; *UAS-sgRNA::castor*; *UAS-Cas9.P2*
Figure 5G-L	Labels adult E-PG neurons and drives UAS constructs in the Type 2 progenitors for:<br>Cas9.P2 control (i)<br>Castor-KO (ii)	Females containing *10xUAS-IVS-myr::smGdP::HA, 13xLexAop2-IVS-myr::smGdP::V5*; *R60D05-LexA*; *Pointed-Gal4* were crossed to males containing either (i) *; ; UAS-Cas9.P2*, (ii) ; *UAS-sgRNA::castor*; *UAS-Cas9.P2*
Figure 6A-F	Labels adult P-EN neurons and drives UAS constructs in the Type 2 progenitors for:<br>LacZ control (i)<br>Castor ME#1 (ii)<br>Castor ME#2 (iii)	Females containing *10xUAS-IVS-myr::smGdP::HA, 13xLexAop2-IVS-myr::smGdP::V5*; *R12D09-LexA*; *Pointed-Gal4* were crossed to males containing either (i) *; UAS-LacZ ;* (ii) ; *UAS-Castor* ; (iii) ; ; *UAS-Castor*

Development • Supplementary information

Figure 6G-L	Labels adult E-PG neurons and drives UAS constructs in the Type 2 progenitors for: LacZ control (i) Castor ME#1 (ii) Castor ME#2 (iii)	Females containing *10xUAS-IVS-myr::smGdP::HA, 13xLexAop2-IVS-myr::smGdP::V5*; *R60D05-LexA*; *Pointed-Gal4* were crossed to males containing either (i) *; UAS-LacZ ;* (ii) ; *UAS-Castor* ; (iii) ; ; *UAS-Castor*
Figure S2A-F	Labels larval Type 2 lineage and drives UAS constructs in the Type 2 progenitors for: Cas9.P2 control (i) Castor-KO#1 (ii) LacZ control (iii) Castor ME#1 (iv) Castor ME#2 (v)	Females containing *10XUAS-IVS-myr::GFP ; Pointed-Gal4* were crossed to males containing either (i) *; ; UAS-Cas9.P2*, (ii) ; *UAS-sgRNA::castor*; *UAS-Cas9.P2* (iii) *; UAS-LacZ ;* (iv) ; *UAS-Castor* ; (v) ; ; *UAS-Castor*
Figure S3A-C	Labels larval Type 2 lineage and drives UAS constructs in the Type 2 progenitors for: Cas9.P2 control (i) Castor-KO (ii)	Females containing *10XUAS-IVS-myr::GFP ; Pointed-Gal4* were crossed to males containing either (i) *; ; UAS-Cas9.P2*, (ii) ; *UAS-sgRNA::castor*; *UAS-Cas9.P2*
Figure S3D-F	Labels larval Type 2 lineage and drives UAS constructs in the Type 2 progenitors for: Cas9.P2 control (i) Svp-KO#1 (ii) Svp-KO#2 (iii)	Females containing *10XUAS-IVS-myr::GFP ; Pointed-Gal4* were crossed to males containing either (i) *; ; UAS-Cas9.P2*, (ii) ; *UAS-sgRNA::svp* (VDRC #341527) ; *UAS-Cas9.P2* , or (iii) ; *UAS-sgRNA::svp* (VDRC #341390) ; *UAS-Cas9.P2*
Figure S4A-C	Labels adult P-FN neurons and drives UAS constructs in the Type 2 progenitors for: Cas9.P2 control (i) Castor-KO#1 (ii)	Females containing *10xUAS-IVS-myr::smGdP::HA, 13xLexAop2-IVS-myr::smGdP::V5*; *R16D01-LexA*; *Pointed-Gal4* were crossed to males containing either (i) *; ; UAS-Cas9.P2*, (ii) ; *UAS-sgRNA::castor*; *UAS-Cas9.P2*
Figure S4D-F	Labels adult E-PG neurons and drives UAS constructs in	Females containing *10xUAS-IVS-myr::smGdP::HA, 13xLexAop2-IVS-myr::smGdP::V5*; *R16D01-LexA*; *Pointed-Gal4* were crossed to males containing

Development • Supplementary information

	the Type 2 progenitors for: LacZ control (i) Castor ME#1 (ii) Castor ME#2 (iii)	either (i) *; UAS-LacZ ;* (ii) ; *UAS-Castor* ; (iii) ; ; *UAS-Castor*

Table S3. Antibodies used.

Antibody	Source and identifier	Additional information
Chicken anti-GFP	Aves: 1020	(1:1000)
Rabbit anti-Castor	PMID:1418995; W. Odenwald	(1:1000)
Mouse anti-Seven-up 6F7	DSHB: Hiromi, Y. / Hondo, T. / Kanda, H.	(1:4)
Rat anti-Deadpan	Abcam: 11D1 BC7.1B	(1:20)
Guinea pig anti-Runt	Claude Desplan lab (NYU)	(1:1000)
Guinea pig anti-Toy	Genscripts U1806FG070_27/DC0126	(1:1000)
Mouse anti-Cut 2B10	DSHB: Rubin, G.M.	(1:10)
Mouse anti-Dac mAbdac1-1	DSHB: Rubin, G.M.	(1:50)
Mouse anti-Elav 9F8A9	DSHB: Rubin, G.M.	(1:50)
Mouse anti-V5 tag	ThermoFisher: R960-25 (previously Invitrogen 46-0705)	(1:1000)
Rabbit anti-V5 tag	Cell signaling: 13202S	(1:1000)
Mouse anti-nc82	DSHB: Buchner, E.	(1:100)
Alexa Fluor® 488 AffiniPure Donkey Anti-Chicken IgY (IgG) (H+L)	Jackson ImmunoResearch, West Grove, PA: 703-545-155	(1:400)
Rhodamine Red™-X (RRX) AffiniPure Donkey Anti-Rat IgG (H+L)	Jackson ImmunoResearch, West Grove, PA: 712-295-153	(1:400)
Alexa Fluor® 647 AffiniPure Donkey Anti-Mouse IgG (H+L)	Jackson ImmunoResearch, West Grove, PA: 715-605-151	(1:400)

Development • Supplementary information

Rhodamine Red™-X (RRX) AffiniPure Donkey Anti-Guinea pig IgG (H+L)	Jackson ImmunoResearch, West Grove, PA: 706-295-148	(1:400)
Alexa Fluor® 488 AffiniPure Donkey Anti-Mouse IgG (H+L)	Jackson ImmunoResearch, West Grove, PA: 715-295-151	(1:400)
Alexa Fluor® 488 AffiniPure Donkey Anti-Rabbit IgG (H+L)	Jackson ImmunoResearch, West Grove, PA: 711-295-152	(1:400)
Rhodamine Red™-X (RRX) AffiniPure Donkey Anti-Mouse IgG (H+L)	Jackson ImmunoResearch, West Grove, PA: 715-295-151	(1:400)
Alexa Fluor® 405 AffiniPure Donkey Anti- Mouse IgG (H+L)	Jackson ImmunoResearch, West Grove, PA: 715-475-150	(1:200)

Table S4. Raw data for experiments done. Each tab shows the raw data for the indicated figure or portion of a figure.

Available for download at
https://journals.biologists.com/dev/article-lookup/doi/10.1242/dev.204318#supplementary-data
