# Supplementary material for: Castor is a temporal transcription factor that specifies early born central complex neuron identity
Source: Development. 2024 Dec 16;151(24):dev204318. doi: 10.1242/dev.204318 (PMC11701512; doi:10.1242/dev.204318)
Supplement: Supplementary file 1 [file develop-151-204318_review_history.pdf]

Castor is a temporal transcription factor that specifies early born central complex neuron identity

Noah R. Dillon and Chris Q. Doe
DOI: 10.1242/dev.204318

Editor: Thomas Lecuit

Review timeline

Original submission:	9 August 2024
Editorial decision:	3 October 2024
First revision received:	18 October 2024
Accepted:	20 November 2024

Original submission

First decision letter

MS ID#: DEVELOP/2024/204318
MS TITLE: Castor is a temporal transcription factor that specifies early born central complex neuron identity

AUTHORS: Chris Q Doe and Noah Dillon

I sincerely apologise for the long delay before getting back to you due to difficulties finding reviewers in the summer, and then due to one reviewer not sending their comments. I have now received two the referees' reports on the above manuscript, and have reached a decision. The referees' comments are appended below, or you can access them online: please go to:

As you will see, the referees express strong interest in your work, but one of them has some significant criticisms and recommends a revision of your manuscript before we can consider publication. If you are able to revise the manuscript along the lines suggested, which should involve further experiments, I will be happy receive a revised version of the manuscript. Your revised paper will be re-reviewed by one or more of the original referees, and acceptance of your manuscript will depend on your addressing satisfactorily the reviewers' major concerns. Please also note that Development will normally permit only one round of major revision. If it would be helpful, you are welcome to contact us to discuss your revision in greater detail. Please send us a point-by-point response indicating your plans for addressing the referees' comments, and we will look over this and provide further guidance.

Reviewer 1

Advance summary and potential significance to field

This (another) solid work by the Doe lab examines the temporal fating mechanisms of the Drosophila type II neuronal lineages which produce postmitotic neurons through transient amplifying precursors resembling primate brain cortical neurogenesis. It demonstrates Castor as a bona-fide temporal transcription factor in the fly type II neuronal lineages. It also addresses the interplay between Castor and Imp/Syp temporal gradients, indirectly through Svp known to trigger the Imp/Syp temporal gradients. Consistent with published works, it reveals no evidence of cross-

© 2024. Published by The Company of Biologists under the terms of the Creative Commons Attribution License (https://creativecommons.org/licenses/by/4.0/).

regulation, implicating parallel (likely serial) Cas and Imp/Syp mechanisms in diversifying temporal fates of fly type II neuronal lineages.

Comments for the author

1. Given the requirement of Cas for E-PG, it is unclear how Svp knockout extends the E-PG production without extending Cas expression. I wonder if epistasis with double svp/cas knockouts can provide additional clues.

2. Extending Cas expression increases P-ENs but not E-PGs that arise from young and old INPs respectively, but not same INPs. I could not remember the evidence indicating they arise from different INPs. Please provide more specific info about this.

Reviewer 2

Advance summary and potential significance to field

Reviewer 1: Understanding how neural diversity is generated is a long-standing question in neurodevelopment. Recent studies on the Drosophila ventral nerve cord, central brain type II neurobasts and optic lobe have revealed that neural progenitors are temporally patterned during development. A series of temporal transcription factors (TTFs) are expressed in neural progenitors, sequentially generating different types of neurons. The Drosophila central complex is known to originate from eight Type II neuroblasts per hemisphere, which further differentiate into multiple different cell types. However, how these different cell types are generated and whether temporal patterning plays a role in Type II lineages remain unclear. Castor (Cas), a transcription factor known to function as a TTF in embryonic VNC lineage, is expressed in early Type II NB lineages. Dillon and Doe investigate whether Cas functions as a TTF in Type II lineages as well. They use the E-PG and P-EN INP lineages in the central complex that are generated at early NB temporal windows. They show that Cas is necessary to specify these two lineages, though it only mildly influences P-EN neurogenesis upon overexpression. This provides a more detailed understanding of Cas function in Type II lineages. However, additional experiments are needed to clarify how Cas controls the production of E-PG and P-EN INP lineages. The authors must also explore the relationship between Cas and the Imp/Syp RNA-binding protein gradients to further elucidate the molecular mechanisms involved. Additionally, they should analyze how Castor differentially influences the three INP sub-lineages generated in its time windows (PF-R, EPG and P-EN and P-FN).

Major concerns
1. Fig. 1A: The authors' model that Castor functions in T2NBs differs from the model previously proposed by Ren et al. (PMID: 28434858). In the current manuscript, the authors suggest that Castor is downstream of Imp and Syp while Ren et al. propose that Castor is upstream of Imp and Syp. Indeed, overexpressing Castor leads to prolonged Imp expression and a shortened Syp expression window. Given that the authors show that Cas and Svp do not have cross-regulation, it is more plausible that Cas and Svp function through the regulation of the Imp and Syp gradient. If the authors wish to argue that Castor is downstream of Imp and Syp, they must provide more evidence. In particular, they must analyze the relationship between Cas/Svp and Imp/Syp, the latter being basically ignored in the current paper.

2. According to the Methods section, the EdU experiment shown in Fig. 1C-E was conducted on larvae reared at 18-22Â°C, whereas flies used in other experiments were reared at 25Â°C. Given that Drosophila exhibits delayed larval development at lower temperatures, the specific timing for E-PG lineage generation might be delayed compared to flies reared at 25Â°C. Is there a specific reason for this difference?

3. Fig. 3E, Fig. 6C, Fig. 6I, Supplemental Fig. 2C, and 2F: The statistical analysis used in these figures involves a one-way ANOVA followed by unpaired t-tests between the control and experimental groups. However, the authors should use a post hoc test (e.g., Dunnett's test) to obtain the p-values for comparisons between each group. Without this, there is an increased risk of making a Type I error, leading to p-values that may be artificially smaller than the true values.

© 2024. Published by The Company of Biologists under the terms of the Creative Commons Attribution License (https://creativecommons.org/licenses/by/4.0/).

4. Fig. 4D: Why do the authors observe that only ~30% of T2NBs are positive for Castor at 24h ALH and that none express it later? Does this mean that T2NBs are not synchronous or that there is variability as to when each T2NB turns on Cas. To check whether the experiments catch the appropriate timing, the authors should look at both sides of the brain, and see whether the same NB expresses, or does not express Cas on the other side? The same observation was made for Svp in Dillon et al. (Fig 2I indicates that fewer that 100% express Svp when integrating all time points)? Or are there other technical challenges that hinder the accurate detection of Castor expression in T2NBs?

5. Fig.4: It is confusing that Castor is transiently expressed in T2NBs around 24h-36h ALH, yet the percentage of Cas+ INPs continues to increase from 24h to 96h ALH. Does this imply that late-born INPs also express Castor? If Castor functions as a temporal transcription factor in T2NBs and its expression is inherited by INPs, one would expect either no change or a gradual decrease in the percentage of Cas+ INPs, as INPs from later temporal windows would dilute the proportion of Cas+ INPs. Or do the authors propose that Castor is reactivated in late-born INPs for a different function unrelated to its role as a TTF?

6. Fig.5F: Upon Castor knockout (KO), the authors are still able to identify some P-EN LexA+ neurons, some of which they describe as "escapers" that did not successfully undergo Castor knockout. However, among these P-EN LexA+ neurons, 50% are double-negative for known P-EN markers. Do these double-negative neurons still project into the EB and NO? It would be interesting to determine if these double-negative neurons belong to another cell type by using other cell type markers, which might have been accidentally captured by the P-EN LexA line.

7. The authors do not provide sufficient evidence to support the claim that Castor is a TTF, as stated in the title and throughout the manuscript. First, Castor and Svp do not exhibit cross-regulation, an important feature of TTFs in several regions of the brain and embryonic VNCs. Second, the phenotype resulting from Cas overexpression appears to be mild for the P-EN lineage and shows no significant effects on E-PG neurons. Therefore, the authors should be much more cautious in stating that Cas is a TTF.

Minor
1. Line 121-123: This paper uses the P-EN and E-PG neuron lineages as a system to investigate whether Castor functions as a TTF, building on the work characterized by Sullivan et al. (PMID: 30706848) and Dillon et al. (PMID: 38230563). However, the introduction does not mention the current understanding of how these two lineages are generated or how they differ in terms of their lineage generation. It would be helpful if the authors could discuss the mechanisms underlying the generation of these two lineages and include a schematic illustration of their birthdates, similar to Fig. 3I in Sullivan et al.

2. Fig.2A-A'': Yellow outline labels the LexA+ neurons, which are E-PG neurons. There are 7 LexA+ neurons in Fig.2A, but only 6 of them are outlined in yellow.

3. Supplemental Fig.2E'': The picture is missing the label for E'' and differs from Supplemental Fig. 2E and Fig. 2E'. This might be due to the image not being rotated as intended.

4. Some of the references need to be fixed, line 557: eLife; line 444, 479, 492, 557: Curr. Biol. instead of Curr. Biol. CB

Reviewer comments and our responses

We thank the reviewers for their thoughtful and constructive comments. We agree with most comments, leading to new experiments with our addition of multiple new and revised figures. In addition, there were several reviewer comments that were due to a lack of clarity on our part; in response we have improved the text to clarify our meaning. Overall, we appreciate the reviewer comments, and they have significantly improved our manuscript.
Reviewer 1

© 2024. Published by The Company of Biologists under the terms of the Creative Commons Attribution License (https://creativecommons.org/licenses/by/4.0/).

Understanding how neural diversity is generated is a long-standing question in neurodevelopment. Recent studies on the Drosophila ventral nerve cord, central brain type II neuroblasts and optic lobe have revealed that neural progenitors are temporally patterned during development. A series of temporal transcription factors (TTFs) are expressed in neural progenitors, sequentially generating different types of neurons. The Drosophila central complex is known to originate from eight Type II neuroblasts per hemisphere, which further differentiate into multiple different cell types. However, how these different cell types are generated and whether temporal patterning plays a role in Type II lineages remain unclear. Castor (Cas), a transcription factor known to function as a TTF in embryonic VNC lineage, is expressed in early Type II NB lineages. Dillon and Doe investigate whether Cas functions as a TTF in Type II lineages as well. They use the E-PG and P-EN INP lineages in the central complex that are generated at early NB temporal windows. They show that Cas is necessary to specify these two lineages, though it only mildly influences P-EN neurogenesis upon overexpression. This provides a more detailed understanding of Cas function in Type II lineages.

Thank you for the accurate summary of temporal pattering in the type II neuroblasts, and its importance in brain development.

However, additional experiments are needed to clarify how Cas controls the production of E-PG and P-EN INP lineages. The authors must also explore the relationship between Cas and the Imp/Syp RNA-binding protein gradients to further elucidate the molecular mechanisms involved. Additionally, they should analyze how Castor differentially influences the three INP sub-lineages generated in its time windows (PF-R, EPG and P-EN and P-FN).

We agree with these comments. We now cite Ren et al (PMID 28434858) where they show in figure 4 that Cas overexpression results in extension of elevated Imp levels. This is consistent with Cas being upstream of Imp (see next point). Our Cas and Syp antibodies are both in rabbit, precluding co-expression studies, but we expect Syp to be low when Imp is high, as has been shown in all systems studied to date. Additionally, the focus of our paper was to investigate whether Castor is a TTF in Type 2 neuroblast lineages, rather than its relationship to other TTFs. Furthermore, previous work has already described the relationship between Castor and Imp/Syp within the lineage (Ren et al. PMID: 28434858). We have added an additional figure S4 from new experiments showing that Castor is not sufficient or necessary for specifying the late born P-FN neuron identity. We have addressed additional concerns below.

Major concerns

1. Fig. 1A: The authors' model that Castor functions in T2NBs differs from the model previously proposed by Ren et al. (PMID: 28434858). In the current manuscript, the authors suggest that Castor is downstream of Imp and Syp while Ren et al. propose that Castor is upstream of Imp and Syp. Indeed, overexpressing Castor leads to prolonged Imp expression and a shortened Syp expression window. Given that the authors show that Cas and Svp do not have cross-regulation, it is more plausible that Cas and Svp function through the regulation of the Imp and Syp gradient. If the authors wish to argue that Castor is downstream of Imp and Syp, they must provide more evidence. In particular, they must analyze the relationship between Cas/Svp and Imp/Syp, the latter being basically ignored in the current paper.

Thank you for catching our error. We now cite Ren et al. (PMID: 28434858) for being the first to show that Cas/Svp are *upstream* of Imp/Syp, and that Cas overexpression can extend high Imp levels. We have replaced the old Figure 1A with a new Figure 1A that properly highlights the fact that Cas/Svp are upstream of Imp/Syp. The corresponding text change is “Castor and Svp act upstream of the Imp and Syncrip gradient (Ren et al., 2017), but whether Castor specifies different neuronal subtypes remains unknown." Line 84-86

2. According to the Methods section, the EdU experiment shown in Fig. 1C-E was conducted on larvae reared at 18-22°C, whereas flies used in other experiments were reared at 25°C. Given that Drosophila exhibits delayed larval development at lower temperatures, the specific timing for E-PG lineage generation might be delayed compared to flies reared at 25°C. Is there a specific reason for this difference?

Yes, we now clarify this point. Prior to EdU, larvae were raised at the standard 25°C. We raised larvae at 18-22°C only *after* we initiated EdU feeding. We found in pilot experiments that larvae

© 2024. Published by The Company of Biologists under the terms of the Creative Commons Attribution License (https://creativecommons.org/licenses/by/4.0/).

feeding on EdU at 25°C leads to high mortality rates. By raising at 25°C until EdU feeding, we remain consistent with the birth dating of these neurons and subsequent experiments. We apologize for not adding these details in the methods and have done so now. “Larvae were raised at 25°C to an appropriate stage before being fed food containing 20 μg/ml EdU nonstop from the initial age feeding started until pupation. Larvae fed on EdU were raised at temperatures between 18°C and 21°C until adults eclosed.” Lines: 375-379

3. Fig. 3E, Fig. 6C, Fig. 6I, Supplemental Fig. 2C, and 2F: The statistical analysis used in these figures involves a one-way ANOVA followed by unpaired t-tests between the control and experimental groups. However, the authors should use a post hoc test (e.g., Dunnett's test) to obtain the p-values for comparisons between each group. Without this, there is an increased risk of making a Type I error, leading to p-values that may be artificially smaller than the true values.

Thank you for the helpful suggestion; we agree and have performed the one-way ANOVAs with a Dunnett’s test for all the post hoc texts (see figure and legend changes). This resulted in only one change: that Cas misexpression construct #2 (Castor-ME#2) and controls were not statistically significant (sFig2F). However, we see biological significance with ectopic Castor expression at 48h with Castor-ME#2 and never in control or WT brains at 48h. Thus, we have chosen to keep Castor-ME#2 in the manuscript.

4. Fig. 4D: Why do the authors observe that only ~30% of T2NBs are positive for Castor at 24h ALH and that none express it later? Does this mean that T2NBs are not synchronous or that there is variability as to when each T2NB turns on Cas. To check whether the experiments catch the appropriate timing, the authors should look at both sides of the brain, and see whether the same NB expresses, or does not express Cas on the other side? The same observation was made for Svp in Dillon et al. (Fig 2I indicates that fewer that 100% express Svp when integrating all time points)? Or are there other technical challenges that hinder the accurate detection of Castor expression in T2NBs?

Thanks for the question. In fact, we see different patterns of Cas expression in brain lobes from the same larvae, indicating that Cas expression is asynchronous. Our observation of asynchronous Castor expression in ~30% of T2NBs at 24h is similar to what was previously reported in Ren et al. (PMID: 28434858).

5. Fig.4: It is confusing that Castor is transiently expressed in T2NBs around 24h-36h ALH, yet the percentage of Cas+ INPs continues to increase from 24h to 96h ALH. Does this imply that late-born INPs also express Castor? If Castor functions as a temporal transcription factor in T2NBs and its expression is inherited by INPs, one would expect either no change or a gradual decrease in the percentage of Cas+ INPs, as INPs from later temporal windows would dilute the proportion of Cas+ INPs. Or do the authors propose that Castor is reactivated in late-born INPs for a different function unrelated to its role as a TTF?

We agree this is an interesting observation. Previous studies only reported expression within the T2NBs (Ren et al PMID: 28434858 and Syed et al PMID: 28394252). We note that many TTFs have additional expression domains that are distinct from their role as TTFs; including INPs and neurons which have distinct Imp expression patterns that are not simply inherited from the parental neuroblast. We published this recently (Munroe et al. PMID 38031099).

6. Fig.5F: Upon Castor knockout (KO), the authors are still able to identify some P-EN LexA+ neurons, some of which they describe as "escapers" that did not successfully undergo Castor knockout. However, among these P-EN LexA+ neurons, 50% are double-negative for known P-EN markers. Do these double-negative neurons still project into the EB and NO? It would be interesting to determine if these double-negative neurons belong to another cell type by using other cell type markers, which might have been accidentally captured by the P-EN LexA line.

We agree that it is interesting to see P-EN LexA expression remain with the absence of the Runt and Cut transcription factors. Unfortunately, we do not have the tools necessary to identify the molecular expression of these LexA positive, double TF negative neurons. Regarding your question of projections to CX neuropils, we do report that remaining P-EN LexA+ neurons as a population still target the PB, EB, and NO (Fig 5B). One possibility is that the remaining LexA+ neurons are partially

© 2024. Published by The Company of Biologists under the terms of the Creative Commons Attribution License (https://creativecommons.org/licenses/by/4.0/).

fate transformed with the loss of Castor which may provide the phenotype we see with a mix molecular identities.

7. The authors do not provide sufficient evidence to support the claim that Castor is a TTF, as stated in the title and throughout the manuscript. First, Castor and Svp do not exhibit cross-regulation, an important feature of TTFs in several regions of the brain and embryonic VNCs. Second, the phenotype resulting from Cas overexpression appears to be mild for the P-EN lineage and shows no significant effects on E-PG neurons. Therefore, the authors should be much more cautious in stating that Cas is a TTF.

We respectfully disagree with the first point and definitely agree with the second. First, regarding cross-regulation -- TTFs are defined by their ability to specify "temporal identity" in multiple lineages... distinct from TFs that generate a specific type of cell (e.g. the TTF Hb specifies first born MN, IN, glia in different embryonic NB lineages, whereas the TF Repo promotes glia identity only). There is no reason that a TTF must cross-regulate with TTFs in an adjacent temporal window. Furthermore, we do not know the TTFs that are expressed immediately before and after the Castor expression window. When these are found, it may reveal cross-regulation.

Moreover, cross-regulation of Castor and Svp is not required for Castor to be classified as a TTF. Svp in both embryonic and larval NB lineages has been determined to be a switching factor and not a TTF (Kanai et al., 2005 PMID: 15691762; Mettler et al., 2006 PMID: 16396905; Maurange et al., 2008 PMID: 18510932; Benito-Sipos et al., 2011 PMID: 22071101; Kohwi et al., 2011 PMID: 21429984; Ren et al., 2017 PMID:28434858; Syed et al., 2017 PMID: 28394252; Dillon et al., 2024 PMID: 38230563). We and others have found it to be strange that Castor and Svp are not cross-regulatory (Ren et al., 2017 PMID:28434858; Syed et al., 2017 PMID: 28394252). However, this makes the Type 2 lineage an appealing system as it will require identifying additional, candidate TTFs to see if a TTF cascade of cross-regulation with Castor is present. We highlight this in our discussion “This raises an open question of whether T2NBs use a TTF cascade or another mechanism of patterning to regulate temporal progression. Additional TTFs will need to be identified to test these hypotheses.” Lines 301-304.

Second, we were also surprised to see our Castor misexpression constructs only yielded mild phenotypes. We have added the text: “One explanation for this could be a limited competency window in which T2NBs can response to prolonged Castor expression. Previous work in the embryonic NB7-1 lineage showed the first TTF, Hunchback, can only produce ectopic early born fates during a limited window due to Svp-independent temporal progression pathways (Kohwi et al., 2011 PMID: 21429984).” Lines 326-330.

Minor

1. Line 121-123: This paper uses the P-EN and E-PG neuron lineages as a system to investigate whether Castor functions as a TTF, building on the work characterized by Sullivan et al. (PMID: 30706848) and Dillon et al. (PMID: 38230563). However, the introduction does not mention the current understanding of how these two lineages are generated or how they differ in terms of their lineage generation. It would be helpful if the authors could discuss the mechanisms underlying the generation of these two lineages and include a schematic illustration of their birthdates, similar to Fig. 3I in Sullivan et al.

We agree and now more clearly introduce the P-EN and E-PG neurons. We have added a lineage scheme depicting the different neuron identities used in this study similar to the Sullivan et al. paper in Fig 1F and added additional text “Our birth dating of CX neurons provide two early born identities, P-EN and E-PG neurons, and one late born identity, P-FN neurons (Fig 1F) (this study; Dillon et al., 2024).” lines 138-140 and “These neuron fates are born in different INP windows with P-EN neurons derived from young INPs and E-PG neurons derived from old INPs (Fig 1F) (Sullivan et al., 2019).” lines 144-146

2. Fig.2A-A": Yellow outline labels the LexA+ neurons, which are E-PG neurons. There are 7 LexA+ neurons in Fig.2A, but only 6 of them are outlined in yellow.

Thank you for pointing this out, we have added the additional annotation to Fig 2A’,A’’.

© 2024. Published by The Company of Biologists under the terms of the Creative Commons Attribution License (https://creativecommons.org/licenses/by/4.0/).

3. Supplemental Fig.2E'': The picture is missing the label for E'' and differs from Supplemental Fig. 2E and Fig. 2E'. This might be due to the image not being rotated as intended.

Thank you for catching this, we have rotated sFig 2 E'' to match the accompanying panels.

4. Some of the references need to be fixed, line 557: eLife; line 444, 479, 492, 557: Curr. Biol. instead of Curr. Biol. CB

Thank you for catching these, we have fixed the references.

Reviewer 2

This (another) solid work by the Doe lab examines the temporal fating mechanisms of the Drosophila type II neuronal lineages which produce postmitotic neurons through transient amplifying precursors resembling primate brain cortical neurogenesis. It demonstrates Castor as a bona-fide temporal transcription factor in the fly type II neuronal lineages. It also addresses the interplay between Castor and Imp/Syp temporal gradients, indirectly through Svp known to trigger the Imp/Syp temporal gradients. Consistent with published works, it reveals no evidence of cross-regulation, implicating parallel (likely serial) Cas and Imp/Syp mechanisms in diversifying temporal fates of fly type II neuronal lineages.

Thank you for the kind words and taking the time to review our work. We have addressed each of your suggestions down below.

1. Given the requirement of Cas for E-PG, it is unclear how Svp knockout extends the E-PG production without extending Cas expression. I wonder if epistasis with double svp/cas knockouts can provide additional clues.

We agree that a double Svp/Cas knockout would be informative to how the Type 2 lineage is patterned. However, our CRISPR toolset with the sgRNAs does not allow us to perform this experiment. We discuss this interesting finding in the Discussion "Interestingly, loss of Svp does not extend the Castor window (Fig. S3) (Ren et al., 2017; Syed et al., 2017) but does extend the production of early born neuron identities (Fig. 3) (Dillon et al., 2024). One explanation for this could be a limited competency window in which T2NBs can response to prolonged Castor expression. Previous work in the embryonic NB7-1 lineage showed the first TTF, Hunchback, can only produce ectopic early born fates during a limited window due to Svp-independent temporal progression pathways (Kohwi et al., 2011). This suggests that additional early factors may be required for specifying fates and could explain the lack of Castor sufficiency to produce additional E-PG neurons." Lines 323-332.

2. Extending Cas expression increases P-ENs but not E-PGs that arise from young and old INPs respectively, but not same INPs. I could not remember the evidence indicating they arise from different INPs. Please provide more specific info about this.

Previous work in the lab published by Sullivan et al 2017 (PMID: 30706848) identified P-ENs arise from young INPs and E-PGs arise from old INPs. We identify in this study and our previous study Dillon et al 2024 (PMID: 38230563) that P-ENs and E-PGs are born within the same T2NB temporal window. We revised and more clearly stated this work in our manuscript with an additional Figure panel (Fig 1F): and text: "These neuron fates are born in different INP windows with P-EN neurons derived from young INPs and E-PG neurons derived from old INPs (Sullivan et al., 2019)." Lines: 144-146.

This study investigates how the various neurons of the Central Complex in the Drosophila brain are made during development. The authors focus on Castor (Cas), a transcription factor known to be expressed along two temporal windows in neuroblasts, in the embryo and early larva. The precise role of Cas in early larval T2 NBs is not clearly defined. Here, the authors confirm the temporal expression pattern of Cas in T2 NBs and find that it is also expressed in INPs and neurons produced by T2NBs. They identify new molecular markers for a subset of central complex neurons produced during the temporal window of Cas expression in T2NBs. Finally, they show that Cas inactivation

© 2024. Published by The Company of Biologists under the terms of the Creative Commons Attribution License (https://creativecommons.org/licenses/by/4.0/).

leads to the absence of such neurons, while Cas misexpression is not sufficient to cause their expansion.

This manuscript is well written. Experiments and results are clear and well described. Altogether, the manuscript suggests an important role of Cas is determining the identity of central complex neurons produced during larval stages by T2NBs. However, I have some concerns that need to be addressed before considering publication. My main issue concerns the fact that the expression pattern of Cas in INPs and neurons is not in line with its narrow expression in T2NBs, suggesting a potential reactivation of Cas in INPs and neurons, uncoupled from the NB, and possibly revealing a different and more general role of Cas in the maturation of neurons.

Thank you for the kind words and taking the time to review our work with helpful suggestions. We have addressed each of your suggestions and concerns down below.

The authors mention their use of sn-RNAseq data from a BioRxiv manuscript (Epiney et al., 2023) to identify new markers for adult E-PG neurons. However, there is no description of how they come to identify Dac in these data. Please explain.

We identified unique transcription factor expression in various columnar neurons in the Epiney paper (in preparation and publicly available dataset on BioRxiv; https://doi.org/10.1101/2023.12.10.571022) including the P-EN neuron Cut expression that we previously published (see Dillon et al 2024, PMID: 38230563). That publicly available dataset and analysis from the Epiney manuscript identified Dac as a potential TF marker in E-PG neurons by its mRNA expression levels.

Fig 3A,B: For clarity, instead of brackets, can you delineate more precisely the cell body region of E-PG neurons? If Svp is a switching factor, how do you explain the defective EB morphology? Shouldn't you see more of the E-PG neurons, without any dramatic effect on neuronal projections? Does it mean that Svp is involved in some aspects of neuronal projection/maturation beyond its role as switching factor?

We use brackets to represent the broad region of where cell bodies are located in the Z-projection which obscures a comprehensive view of each individual cell body (Fig 3 A,B). In addition, we use sub panels of confocal slices to more clearly show these cell bodies and outline them in yellow (Fig 3C-D''').

Our previous reporting of Svp-KO in P-EN neurons showed significant disruptions to the CX neuropils (Dillon et al 2024, PMID: 38230563). Thus, we were not surprised to see a defect in E-PG neuron targeting (Fig 3B; sFig1). We have added the following to our discussion to highlight this finding: "We show in this work (sFig1), and previous work (Dillon et al., 2024), that loss of Svp disrupts CX neuropils (Fig. 3, Fig. S1). Surprisingly, the extended production of early born neurons maintain their normal molecular identities of Dac+ Toy+ E-PG neurons and Cut+ Runt+ P-EN neurons (Fig. 3, Fig 7B) (Dillon et al., 2024). This suggests that additional, unknown factors may be regulating CX development outside of the temporal patterning downstream of Svp. Alternatively, late born neuron fates may be required for appropriate CX morphology and loss of these fates leads to abnormal CX architecture." Lines 306-313

Figure 4: It is surprising that Cas is expressed so transiently in T2NBs during early larval stages, but is present in a majority of INPs until late larval stages, as if there were no direct correspondence between expression in NBs and INPs. Are the Cas+ INPs in at 96 hrs all produced by the Cas+ T2NBs before 48 hrs? This could be tested by tracking early-born INPs with EdU.

We agree this is an interesting question given previous studies only reported expression within the T2NBs (Bayraktar and Doe PMID:23783519; Ren et al PMID: 28434858; Syed et al PMID: 28394252). We note that many TTFs have additional expression domains that are distinct from their role as TTFs; and this is likely true for Castor in INPs and neurons. It remains for future work to determine the role of Castor in the INPs and post-mitotic larval neurons. Characterizing the Cas+ INPs and neurons is a huge job that is beyond the scope of this TTF-focused paper.

© 2024. Published by The Company of Biologists under the terms of the Creative Commons Attribution License (https://creativecommons.org/licenses/by/4.0/).

Given that Cas is expressed in T2NBs in an early larval temporal window that is presumably Imp+ and Syp-, all the Cas+ neurons and INPs generated from Cas+ T2NBs should be Imp+ Syp-. Please show these immunostainings to confirm your conclusions.

We published this recently that INPs and neurons have distinct Imp expression patterns that are not simply inherited from the parental neuroblast. (Munroe et al. PMID 38031099). Thus, Imp expression in the INPs and neurons would not be an accurate marker of T2NB temporal birth. Unfortunately, our Castor and Syncrip antibodies are within the same host species so we are unable to look at those dynamics.

Loss of Svp triggers an expansion of E-PG neurons but not an expansion of the Cas temporal window in T2NBs. How do you reconcile with the fact that Cas is required for E-PG specification?

We agree that this is an interesting question and have added in our Discussion: "Interestingly, loss of Svp does not extend the Castor window (Fig. S3) (Ren et al., 2017; Syed et al., 2017) but does extend the production of early born neuron identities (Fig. 3) (Dillon et al., 2024). One explanation for this could be a limited competency window in which T2NBs can response to prolonged Castor expression. Previous work in the embryonic NB7-1 lineage showed the first TTF, Hunchback, can only produce ectopic early born fates during a limited window due to Svp-independent temporal progression pathways (Kohwi et al., 2011). This suggests that additional early factors may be required for specifying fates and could explain the lack of Castor sufficiency to produce additional E-PG neurons." lines: 323-332

Are late born neurons produced upon Cas inactivation. What is the final structure of the central complex? Is it absent?

Excellent questions. We have performed additional experiments to address them. The late born neurons, such as the P-FN neurons, are not affected by the loss of Castor or misexpression of Castor. The CX neuroarchitecture remains intact with all four neuropils present with the loss of Castor. We have added supplementary figures showing these results. sFig. 4, sFig 5, and accompanying text in the new results section "Castor is not necessary or sufficient to specify late born P-FN adult neuron identities" lines: 244-255

A recapitulatory scheme of the T2NB lineage showing when and by which INPs, P-EN and E-PG neurons are made during larval development would be helpful. It could include Cas and Svp expression pattern as well as phenotypes of cas or svp inactivation.

We agree and have added a new summary figure (Fig 7) to clearly show Cas/Svp expression windows, the subsequent lineages, and birth dating relationship between the CX neurons. We have added additional text to clarify these points. "Our birth dating of CX neurons provide two early born identities, P-EN and E-PG neurons, and one late born identity, P-FN neurons (Fig 1F) (this study; Dillon et al., 2024)." lines: 138-140 and "These neuron fates are born in different INP windows with P-EN neurons derived from young INPs and E-PG neurons derived from old INPs (Fig 1F) (Sullivan et al., 2019)." lines: 144-146

First revision

<u>Author response to reviewers' comments</u>

We thank the reviewers for their insightful comments and suggestions to improve the clarity of the manuscript. We have addressed specific questions in individual responses below:

<u>Reviewer 1:</u>

1. Figure 1B: please provide higher magnification images (as insets), as it is not clear that the cell phenotype of day 34 or 50 resembles the CT29 (control) line. The same comment is relevant to Figure 3A.

© 2024. Published by The Company of Biologists under the terms of the Creative Commons Attribution License (https://creativecommons.org/licenses/by/4.0/).

The higher magnification images have been provided as requested and updated in Figures 1B and 2A.

2. *Figure 5H (heatmap): are there 2 clusters of EVTs?*

Yes, there are two clusters which reflect differences in EVT cell differentiation among the chorionic villus-derived cell lines. We now provide differential gene expression analysis for the individual chorionic villus-derived TS cell lines (K01, K09, K23, and K24) compared to the CT-27/CT-29 cell lines. Data provided as new Supplementary Tables 6-13. We have also added an additional Figure that shows correlation plots for individual cell line comparisons (Supplementary Figure 1) and text in the results section (Lines 216-221).

3. *Were there any gene products that did not match the expected differentiation-related changes, based on the control TS cells?*

There were some cell line differences in gene expression. Overall, the correlation of gene expression for the cytotrophoblast versus chorionic villus derived lines were excellent. Datasets for RNA-sequencing from each TS cell line are provided (Supplementary Tables 6-13) and can be inspected for individual gene differences. We are also showing correlations of each of the new chorionic villus lines with the CT-27/CT-29 lines (Supplementary Figure 1).

4. *The culture of TS cells under standard conditions may indeed "normalize" some of the differences among the TS lineages. Did the authors detect differential expression patterns early in culture that were "equalized" over time?*
We did not perform characterization of the new chorionic villus-derived TS cell lines at different stages during the establishment of the lines.

5. *Statistical analysis: was post hoc testing done for multiple comparisons (ANOVA, etc.)?*

No post hoc testing was performed as these were pairwise analyses.

6. *The discussion may benefit from some revision. On one hand, it is too long. For example, lines 231-250 can be shortened to one sentence.*

The introductory paragraph to the Discussion (lines 224-243) provides important insights into the progression of research strategies for investigating trophoblast cells in vitro and has merit.

On the other hand, the authors should consider adding some brief commentary on the potential use of the patient-specific TS lines.

In lines 305 to 310 we describe the utility of the chorionic villus-derived cell lines.

After all, chorionic villus sampling is infrequently used nowadays. What would be typical investigative or clinical reasons to obtain such biopsies and create the TS lines from them?

We understand the reviewer's concern; however, this manuscript does not represent an appropriate forum for commenting on the clinical utility of chorionic villus biopsies.

7. *Trophoblasts from Down syndrome are believed to have aberrant differentiation. As TS cells were derived from a participant with Tri-21 (line K08), It would add to the manuscript's quality if relevant differentiation markers were examined and included in this text.*

We have the requested data for the K08 cell line; however, we are hesitant to include data from a single Down Syndrome TS cell line (n=1). Our concern is that the data may be used <u>inappropriately</u> to reflect the behavior of <u>all</u> Down Syndrome TS cell lines. Consequently, we are revising the text and table to only reflect the chorionic villus-derived TS cell lines we have characterized for this manuscript.

© 2024. Published by The Company of Biologists under the terms of the Creative Commons Attribution License (https://creativecommons.org/licenses/by/4.0/).

8. *(Minor): In the methods, the authors may consider merging the section on isolation and qPCR of mRNA and miRNA.*
The modification has been made as requested (Lines 435-443).

Reviewer 2:

1. *The report opens up the field to exploring trophoblast with aneuploidies but this was not performed. If this could be done it would be a much better paper. 2 samples are Trisomy 18, 1 Trisomy 21 and 2 translocations. There is an opportunity to study here why pregnancies with trisomies and other aneuploidies have abnormal function (Shahbazi 2020, Sugimoto 2022, Wang 2024, Adams 202, Bustos 2020, 2021).*

At this juncture, we do not have sufficient sample sizes (number of independent lines) to make conclusive statements about any of the aneuploidies. Inclusion of such information from a single cell line (n=1) with the idea that they would be representative of a specific aneuploidy would be especially problematic.

2. *In addition, there are previous reports using chorionic villus samples to grow trophoblast but these have generated primary organoids (Schaffers 2022) which more closely resemble normal in vivo trophoblast (Shannon 2024 - not cited). This was not attempted here but would again be good to see.*
Organoids are a potentially useful in vitro model but were not investigated in this report. We are aware of the efforts of Schaffers et al. 2022 and cited the report in the manuscript (line 275). Investigating the establishment of organoids from the chorionic villus-derived trophoblast stem cell lines is a direction that could be pursued in the future.

3. *The success rate is low at 42% Is this related to the gestational age 9 weeks (mostly 12 weeks and earliest 10 weeks)? Haider 2018 reported that they could not generate trophoblast organoids from samples more than 10 weeks gestation.*

We do not have an explanation for the success rate we achieved. Some insights about our success rate were provided in lines 134-140.

4. *The TSC have been tested for trophoblast identity using criteria of Lee 2016 by looking at expression of ELF5 methylation and C19MC. The latter needs a positive control (JEG or JAR).*

Our positive control for this experiment was TS cells derived from cytotrophoblast (CT-27 and CT-29 lines). We do not view the inclusion of JEG or JAR choriocarcinoma cells (cancer cells) as controls is meritorious for the report.

5. *A defining characteristic of trophoblast is HLA expression which differs between villous and extravillous trophoblast. This needs to be performed because others have shown that TSC grown in 2D do not show the normal trophoblast HLA expression (Shannon 2024, Sheridan 2021). What is the pattern of HLA expression in their lines?*

We show that differentiated EVT cells, but not stem state cells, express HLA-G at the transcript and protein levels (Fig. 4B, 4D) in TS^{CT} and TS^{CV} lines. HLA transcripts were measured in all TS^{CT} and TS^{CV} lines by RNA sequencing and presented in Supplementary Tables 2-13. Overall, HLA transcript levels in each cell state were similar between TS^{CT} and TS^{CV} lines. The most abundant HLA transcript in any cell state was *HLA-G* in EVT differentiated cells, which showed an average log2 fold change of ~5 in both TS^{CT} and TS^{CV} cells. *HLA-E* and *HLA-C* were the second and third most highly expressed (also in EVT cell state) based on read counts. *HLA-A* and *HLA-B* had low base mean read counts overall.

Soncin and colleagues (Stem Cell Reports 17:1-15, 2022) investigated the utility of using HLA expression as a defining characteristic of trophoblast cells. They found that HLA expression is affected by culture conditions, especially the inclusion of valproic acid, which is a constituent of the human TS cell culture system used in our experimentation. Consequently, we did not view HLA expression as a definitive indicator of the trophoblast cell phenotype and did not include further HLA analysis in our characterization of the chorionic villus- derived TS cell

© 2024. Published by The Company of Biologists under the terms of the Creative Commons Attribution License (https://creativecommons.org/licenses/by/4.0/).

lines.

6. Fig. 5H: Unlike Fig. 5G, where the three cell states were clearly separated, Fig. 5H shows CVK23 and CVK24 distinct from other cell lines. For instance, there are two clades for STB, with STB from CVK23 and CVK24 separated from the rest. Unexpectedly, EVT from these two cell lines cluster with STB, separating from other EVT. They also exhibit much higher miRNA expression in Fig. 2B. Why are these two cell lines different?

This is an important point. We observed some differences in the chorionic villus derived cell lines, especially regarding their behavior in differentiating into the EVT cell lineage. We now provide RNAseq datasets (Supplementary Tables 6-13) for each chorionic villus-derived cell line maintained in the stem state versus the differentiation states (STB and EVT), which should provide more transparency regarding the transcriptomes of each line. We do not have an explanation why the new chorionic villus-derived TS cell lines exhibit variability among some parameters. This variation among human trophoblast stem cells is likely important and has been previously noted as presented in the Discussion (see lines 279 to 289).

7. The authors measured the transcriptomes of four cell lines for TSCV (TSCVK01, TSCVK09, TSCVK23, and TSCVK24), all with clinically normal karyotypes. Firstly, it would be helpful if the transcriptomes of cell lines from abnormal karyotypes are also measured to investigate the differences between normal and abnormal conditions. Secondly, two of the four cell lines measured exhibited mosaicism (TSCVK09 and TSCVK23). Is this reflected on the transcriptomes?

We have the requested data for the cell lines with abnormal; however, we are hesitant to include data from a TS cell line with a unique karyotype (each would effectively be n=1). Our concern is that the data may be used inappropriately to reflect the behavior of all TS cell lines with a given karyotype. Consequently, we are revising the text and table to only reflect the chorionic villus-derived TS cell lines we have characterized for this manuscript.

We now provide RNAseq datasets (Supplementary Tables 6-13) for each chorionic villus-derived cell line maintained in the stem state versus the differentiation states (STB and EVT), which should provide more transparency regarding the transcriptomes of each line. We cannot make any conclusions about the impact of the mosaicism on transcriptomes for the new chorionic villus-derived TS cell lines.

8. Is there any difference in the transcriptomes of male and female TSC (Gong 2018)?

Based on the work of Sun et al. (J Clin Endocrinol Metab 105:e4831-4847, 2020), there are likely differences in the transcriptomes of male versus female trophoblast cells; however, our sample size is too small to make any definitive statements about the impact of sex on trophoblast stem cell phenotypes.

9. The authors seem to be suggesting that normal karyotypes can turn abnormal after culture. Is this related to the somatic mutational load see by trophoblast at term? This might be explored farther.

In the Discussion (lines 291-303), we offered potential explanations of discrepancies in the karyotypes of the clinical genetic analysis of the chorionic villus biopsy and the karyotype of the TS cell line established from the chorionic villus biopsy. We discussed the possibility of placental mosaicism or a response to the culture conditions. We do not have anything else to present.

Second decision letter

MS ID#: DEVELOP/2024/204318

MS TITLE: Castor is a temporal transcription factor that specifies early born central complex neuron identity

© 2024. Published by The Company of Biologists under the terms of the Creative Commons Attribution License (https://creativecommons.org/licenses/by/4.0/).

AUTHORS: Chris Q Doe and Noah Dillon

I am happy to tell you that your manuscript has been accepted for publication in Development, pending our standard publication integrity checks.

© 2024. Published by The Company of Biologists under the terms of the Creative Commons Attribution License (https://creativecommons.org/licenses/by/4.0/).
